# Ultrasound-guided “short” midline catheters for difficult venous access in the emergency department: a retrospective analysis

**DOI:** 10.1186/s12245-016-0100-0

**Published:** 2016-02-04

**Authors:** Giancarlo Scoppettuolo, Mauro Pittiruti, Sara Pitoni, Laura Dolcetti, Alessandro Emoli, Alessandro Mitidieri, Ivano Migliorini, Maria Giuseppina Annetta

**Affiliations:** Department of Infectious Diseases, Catholic University Hospital, Largo A. Gemelli, 8, 00168 Rome, Italy; Department of Surgery, Catholic University Hospital, Rome, Italy; Intensive Care Unit, Catholic University Hospital, Rome, Italy; Department of Oncology, Catholic University Hospital, Rome, Italy; Coronary Care Unit, Catholic University Hospital, Rome, Italy

**Keywords:** Peripheral intravenous access, Ultrasound guide, Emergency department, Polyurethane catheters, Midline catheters

## Abstract

**Background:**

Acutely ill patients admitted to the emergency department (ED) constantly require at least one fast and reliable peripheral intravenous (PIV) access. In many conditions (morbid obesity, underweight state, chronic diseases, intravenous drug abuse, adverse local conditions, etc.), PIV placement may be challenging.

Ultrasound guidance is a useful tool for obtaining a peripheral intravenous access in the emergency department, particularly when superficial veins are difficult to identify by palpation and direct visualization, though standard peripheral intravenous cannulas are not ideal for this technique of insertion and may have limited duration.

Long polyurethane catheters inserted with ultrasound guidance and direct Seldinger technique appear to have several advantages over short cannulas in terms of success of insertion and of duration.

**Methods:**

A retrospective analysis was conducted on all the ultrasound-guided peripheral venous accesses obtained by insertion of long polyurethane catheters in patients admitted to the emergency department of our university hospital during 1 year. The main indication to the procedure was the urgent need of a peripheral venous access in patients with superficial veins difficult to palpate and/or visualize. All relevant data concerning the insertion and the maintenance of these peripheral lines were collected from the chart.

**Results:**

Seventy-six patients were included in this review. The success rate of insertion was 100 %, with an average of 1.57 punctures per each successful cannulation. The mean time needed for the complete procedure was 9.5 min. In 73 % of patients, the catheter was used for more than 1 week; a minority of catheters were removed prematurely for end of use. No major infective or thrombotic complication was reported.

**Conclusions:**

In our experience, 8- to 10-cm-long polyurethane catheters may offer a fast and reliable peripheral venous access in the emergency department, if placed by ultrasound guidance and with the Seldinger technique. Further studies with prospective, randomized, and controlled design are warranted to confirm our results.

## Background

Acutely ill patients admitted to the emergency department (ED) constantly require at least one fast and reliable peripheral intravenous (PIV) access. Prompt PIV placement is crucial in ED patients, since it allows the administration of urgent and sometimes life-saving medications, though many of these patients may have problems in the identification of superficial veins due to edema or hypovolemia, so that PIV placement can be challenging. Morbid obesity, underweight state, chronic diseases, intravenous drug abuse, and adverse local conditions are other important causes of failure to obtain PIV in this setting [1, 2].

In such cases, alternative routes of venous access include intra-osseous needle placement or central venous cannulation. Intra-osseous access is expensive and not constantly available; also, it lasts for a short period of time (typically few hours), and it must be removed anyway within 24–48 h. Central venous cannulation—particularly in emergency—carries some risk of complications, particularly in patients with poor coagulation status [3–5] and can be time-consuming [6]. Furthermore, current guidelines recommend that central venous catheters (CVCs) inserted in an emergency setting should be removed as soon as possible (within 24–48 h) due to the risk of infection.

Today, the increased availability of portable ultrasound (US) machines has made ultrasound-guided peripheral venous access an increasingly popular option.

In fact, the adoption of US-guided PIV can prevent unnecessary insertions of CVC and the consequent catheter-related complications. In this setting, US-guided peripheral access could not only be safer and faster than an unnecessary central line but also more cost-effective. Moreover, in patients with difficult access, US-inserted PIVs, compared with traditional PIVs, have many proven advantages in terms of rapidity of access, success rates, and patient satisfaction [6–10].

However, recent studies have also highlighted two main disadvantages associated with the placement of US-guided PIVs, when compared to traditional PIVs: a brief duration of the access and some technical difficulties during insertion, related to the “cannula-over-needle” technique [11–13].

Our recent experience suggests that ultrasound insertion of polyurethane catheters slightly longer than traditional peripheral cannulas may have specific advantages in terms of success rate and mean duration of the line, particularly if they are inserted by the direct Seldinger technique.

Recently, peripheral cannulas of intermediate length between PIVs (3.5–5.2 cm) and “standard” midline catheters (15–25 cm) have been introduced in our clinical practice. Though the terminology of these venous-access devices is not yet well defined, they obviously differ from the standard midlines not only because of their length (6–15 cm) but also because of their technique of insertion (direct Seldinger technique, as opposed to the modified or “indirect” Seldinger technique) and their more affordable price (in our hospital, they cost one fifth to one sixth of the price of a midline catheter).

These devices have been called “short midlines” or “long peripheral cannulas” or—sometimes—they have been considered as midlines, without further differentiation. Still, from the clinical point of view, they share some characteristics of PIVs (low cost, rapid insertion) and some of midlines (ultrasound venipuncture, biocompatible material, long duration). In particular, the combined adoption of ultrasound venipuncture and direct Seldinger technique accounts for a faster and simpler insertion if compared to standard midlines and makes them quite appropriate in emergency situations.

We reviewed retrospectively our experience with this new approach to the difficult venous access in the ED, i.e., the ultrasound-guided insertion of 8–10-cm-long polyurethane catheters in peripheral veins of the upper limb using the direct Seldinger technique.

## Methods

This study is a retrospective analysis conducted over patients admitted to the ED of our university hospital during the year 2013. Data collection was based on a retrospective review of the clinical charts.

We hypothesized that the insertion of 8–10-cm polyurethane catheters by direct Seldinger technique might have increased the success rate and the mean lifetime of the line. The Seldinger technique is associated with an easier venous cannulation if compared to the “cannula-over-needle” technique of traditional PIVs. Some technical features, such as the material (polyurethane being more biocompatible than teflon, which is the standard material for short cannulas) and the relevant length of the catheter (which reduces the risk of dislocation) should be associated with a longer duration.

**Fig. 1:**
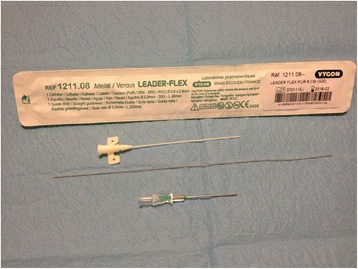
Eight-centimeter polyurethane catheter + 20G needle + 20-cm straight tip guide wire

We have been using polyurethane catheters (Leaderflex, Vygon) available both as 18G or 20G and marketed for both venous or arterial cannulation. The 18G catheter kit includes a 10-cm-long catheter (external diameter 4Fr, internal area 18G), a 54-mm-long 19G needle and a 30-cm straight tip guide wire. The 20G kit (Fig. 1) includes a 8-cm-long 20G catheter, a 38-mm-long 20G needle, and a 20-cm straight tip guide wire. Both catheters are meant for insertion via the direct Seldinger technique, either with or without ultrasound guidance.

All polyurethane catheters had been positioned by specifically trained ED nurses or physicians, according to a specific, standardized protocol of insertion which can be summarized as follows.

**Fig. 2:**
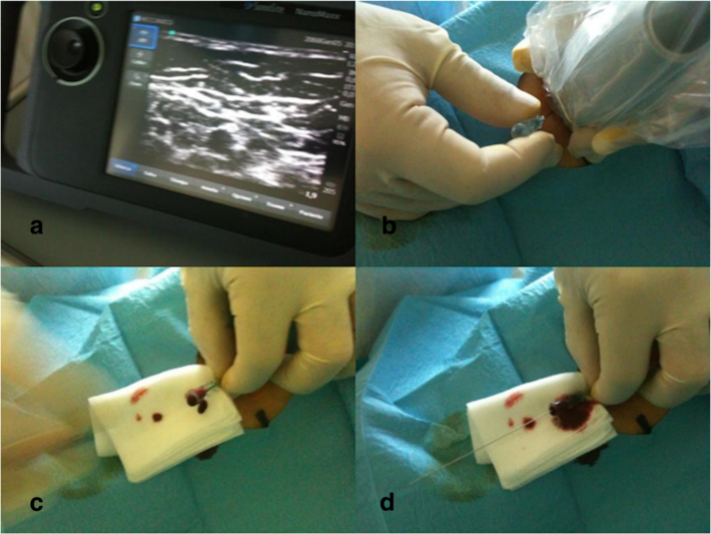
**a** US identification of the vein in short axis. **b** US-guided “out-of-plane” venipuncture. **c** Successful venipuncture is confirmed by blood reflux. **d** Guide wire threaded through the needle

**Fig. 3:**
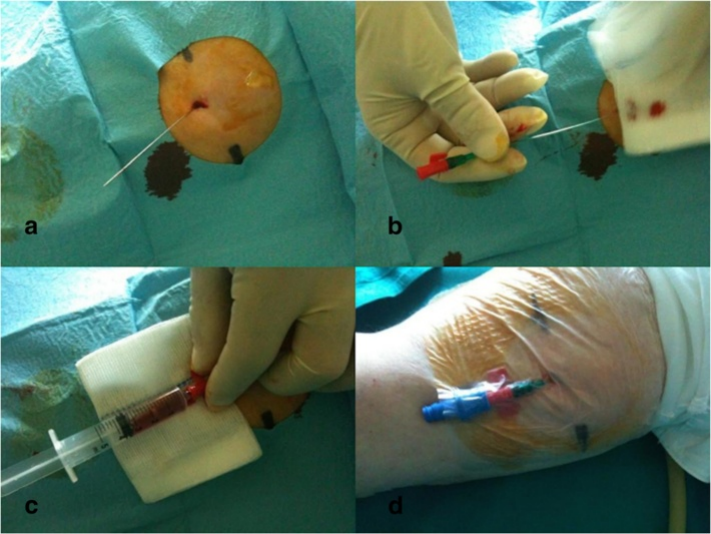
**a** Removal of the needle. **b** Insertion of the catheter over the guide wire. **c** Intravenous placement confirmed by blood return. **d** Final dressing

The patient forearm is supinated and examined with ultrasound so to identify the most appropriate vein to cannulate in terms of position, diameter, and depth (Fig. 2a). Only veins with diameter >3 mm and depth <30 mm from the skin are considered. Upper mid-arm veins (specifically, basilic and brachial veins) are preferred to veins below the elbow. The tourniquet is placed above the elbow, very close to the axilla. The puncture site is scrubbed with 2 % chlorhexidine antiseptic swabs and protected by a sterile fenestrated drape. A portable ultrasound device (NanoMax, Sonosite) with a 5–10-MHz linear probe is commonly used; sterile gel and a sterile cover for the probe are used. The 20G or 18G kit is chosen mostly on the basis of the vein diameter, considering that the 18G catheter (4Fr) requires a vein diameter equal or superior to 4 mm (12Fr). Local anesthesia (1–2 ml of 0.75–1 % ropivacaine) may be needed in the site of insertion, just before the puncture, depending on the patient’s conditions. The vein is punctured under direct ultrasound guidance (vein visualized in short axis, “out-of-plane” puncture) (Fig. 2b, c), and the guide wire is introduced into the needle (Fig. 2d). As the needle is removed (Fig. 3a), the catheter is advanced into the vein, over the guide wire, according to the direct Seldinger technique (Fig. 3b, c). Successful cannulation is confirmed by drawing blood. The access is capped with a needle-free connector, flushed with saline and secured with a sutureless device and/or a transparent semipermeable dressing (Fig. 3d). At the end of each procedure, the most important data regarding the insertion (insertion site, diameter and depth of the vein, duration of the procedure, insertion-related complications) are entered in a specific form which is attached to the patient’s chart.

The study population consisted of all adult patients (>18 years old) admitted to the ED from January 2013 to December 2013, who required the ultrasound-guided insertion of a long polyurethane catheter in the deep veins of the arm. The typical candidate to this procedure was the ED patient with clinical indication to a peripheral venous access but “difficult” superficial veins, i.e., absence of visible and/or palpable vein of the arms or failure of two or more puncture attempts. Obviously, ED patients with clinical indication to central venous access (typically, hemodynamic instability requiring rapid fluid repletion and/or continuous infusion of inotropic drugs) were not candidate to US-guided PIV.

For each case, several data were recorded, concerning both the patient (gender, age, BMI, primary diagnosis) and the venous access (insertion-related complications, success at insertion, number of attempts before insertion, length of the procedure, insertion site, diameter and depth of the vessel, duration of the access, late complications).

All data were included in a software-operated database and analyzed by standard descriptive statistics. Values are reported as the mean ± standard deviation.

According to the policy of our hospital, retrospective studies do not require approval from the Ethics Committee and do not require informed consent from the patient.

## Results

Between January 1st and December 31st, 2013, 76 long peripheral catheters have been inserted in the ED, using US guidance and direct Seldinger technique, in 76 patients (36 males, 40 females).

The mean age was 59.35 ± 19.09 years with a range between 19 and 91. Body mass index was below 30 in 34 patients (44 %). In 31 patients (40.7 %), edema of the subcutaneous tissue was the main cause of difficult venous access.

The primary diagnosis was known neoplastic disease (23 pts), cardiac failure (12 pts), acute lung infection (10 pts), chronic obstructive pulmonary disease (7 pts), decompensated diabetes (5 pts), hepatic cirrhosis (4 pts), urinary tract infection (2 pts), stroke (2 pts), or other chronic diseases (11 pts).

In all patients, catheters were inserted without complication and the success rate of insertion was 100 %. The mean number of attempts before success was 1.57 ± 0.63 (range 1–3). The mean time required by the procedure was 9.5 ± 2.27 min (range 7–16).

We inserted 11 (14 %) 18G catheters and 65 (86 %) 20G catheters. The mean insertion time was not affected by the choice of 18G vs. 20G catheter.

The venipuncture sites were as follows: 34 catheters (45 %) were placed in the basilic vein, 17 (22 %) in a brachial vein, 11 (15 %) in the cephalic vein, and 14 (18 %) were inserted in veins of the forearm.

The mean diameter (mm) of the cannulated vein was as follows: 4.17 ± 0.83 (4–6) for the basilic vein, 4.11 ± 0.48 (3–5) for the brachial veins, 3.81 ± 0.60 (3–5) for the cephalic vein, and 3.57 ± 0.50 (3–4) for the veins of the forearm.

The mean depth (mm) of the cannulated vein was as follows: 10.59 ± 6.15 (3–25) for the basilic vein, 11.82 ± 4.43 (3–17) for the brachial veins, 14.36 ± 5.00 (4–21) for the cephalic vein, and 10.28 ± 2.49 (5–13) for the veins of the forearm.

The catheters were used for several purposes such as administration of fluid and medications; in 40 patients, they were used also for power injection of contrast medium during radiologic exams.

Most catheters (55 patients—73 %) were electively removed after 7–8 days and replaced by more permanent venous-access devices. In a minority of patients (21 patients—27 %), the catheter was removed prematurely for end of use; in this group of patients which had the catheter for less than 1 week, the mean duration was 2.33 ± 2.95 days (range 2–6). No major infective or thrombotic complications were reported. No accidental dislocation occurred.

## Discussion

Ultrasound-guided peripheral venous access has been demonstrated to be superior to the traditional landmark and palpation approach in achieving successful venous cannulation, since it reduces the number of percutaneous punctures and decreases the overall time of the procedure [7, 10, 14, 15]. US-guided peripheral access has also been shown to increase patient satisfaction [6, 15, 16] and prevent the insertion of unnecessary CVCs in the ED [16, 17]. Recent international recommendations have also advocated US guidance for all venous-access sites. In particular, US guidance has been recommended in both pediatric and adult patients for the placement of PIV when a difficult access is anticipated [18].

Literature published during the last decade has stressed two main concerns associated with the US-guided insertion of PIVs.

First, peripheral lines placed under US guidance have a shorter duration than those inserted using the traditional technique [8, 10, 18]. Keyes and colleagues noted an 8 % failure rate in the first hour after US placement of PIVs, as well as a very high rate of dislocation in the first 24 h [10]. Premature end of catheters’ lives was also found by Dargin and colleagues, who reported a 47 % failure rate in the first 24 h [19].

Second, the success rate of US insertion of PIV is not 100 % and seems to be strictly dependent on vessels’ characteristics: success was more likely for larger veins (>0.4 cm) and for vessels at moderate depth (equal or less than 1.5 cm) [11, 12].

In our hypothesis, peripheral polyurethane catheters may overcome these two problems, since they seem to be associated with prolonged duration of the access and higher success rate at insertion.

First, these polyurethane catheters are 8 or 10 cm long, while most traditional peripheral cannulas are 3.5–5.2 cm long. Longer than standard PIV catheters have already been tested by Mills and colleagues: they found out that the insertion of 15-cm-long cannulas into the brachial or basilic veins resulted in a reduced rate of dislocation [8]. However, they still reported a fairly short catheter life (median duration of access was 26 h).

In the present study, long peripheral catheters were associated with a remarkably long duration (more than 1 week), which could be due not only to the length of the catheter but also to the material. Polyurethane is considered to be more biocompatible than teflon, being associated with a reduced incidence of phlebitis, bacterial adherence, and mechanical distortion [20, 21].

As mentioned above, non-US-guided short cannulas have been associated with several local complications such as catheter-related thrombophlebitis, infiltration, and site infection without bacteremia [10, 22–24]. We did not detect any relevant local complication due to our polyurethane catheters. US-guided peripheral lines appear to have a low rate of long-term complications [25, 26]. Also in recent studies on ICU patients, which often have more severe complications than ED patients, very low rates of infiltration (3.4 %), inadvertent removal (2.7 %), and phlebitis (0.7 %) were reported [27, 28].

Second, our success rate was 100 %, and insertion-related complication was none. This is partly related to the advantages of the Seldinger technique—used in our polyurethane catheter—vs. the “cannula-over-needle” technique—used with short cannulas, though a relevant role was also played by the specific experience of our ED team, who had undergone extensive training in US-guided insertion of different venous-access devices (PICCs, midline catheters, and peripheral cannulas). It is interesting that our results have been achieved in a series of catheters inserted by different operators, thus demonstrating the repeatability of these outcomes.

In conclusion, our data suggest that when an emergency peripheral line is needed in patients with difficult venous access, ultrasound-guided placement of long polyurethane peripheral catheters using the Seldinger technique is easy, fast, safe, and cost-effective. In this regard, as a matter of fact, it should be stressed that the average cost of one complete kit including the 8–10-cm catheter, the needle, and the guide wire is €15–€20, which is lower than the cost of a standard CVC, midline or PICC kit. The cost of a short cannula is obviously lower, but the high rate of local complications and the short duration limit its cost-effectiveness.

The main limit of our study lies in its retrospective design. No control group was available to compare US-guided long peripheral access vs. US-guided short peripheral cannulas vs. non-US-guided “traditional” short cannulas.

Other possible limitations of the study are the limitations still inherent in a retrospective design: some late complications may have not been recorded in the patient’s chart; some biases may exist in the selection of the patients in the emergency room (operators particularly skilled in US-guided placement may have decided to place an US-guided peripheral catheter more promptly than less skilled operators).

Also, we cannot offer useful data about the learning curve for this maneuver, since all the ED nurses and physicians who inserted the catheters were already trained in ultrasound-guided insertion of standard midline catheters and PICCs, which requires similar audio-visual skills.

Finally, our sample cannot be considered entirely representative of the ED patient population, as the most severely ill hypovolemic and hemorrhagic patients were excluded since they needed a large bore line for rapid fluid repletion. In this regard, we are currently investigating the US-guided insertion of short (6.4 cm), large bore (8Fr) introducers which are more appropriate for the rapid infusion of fluids.

## Conclusions

Ultrasound guidance is a useful tool to obtain a fast and reliable peripheral intravenous line in ED patients with difficult venous access, thus saving time and avoiding unnecessary CVCs and their potential complications.

US-guided placement of long (8–10 cm) polyurethane catheters by the Seldinger technique seems to be preferable to US-guided placement of short (3.5–5.2 cm) teflon cannulas, in terms of insertion success, early and late complication, and duration of the peripheral line.

Prospective studies with randomized and controlled design are needed to confirm these results on a larger scale.

## Abbreviations

CVCs: central venous catheters
ED: emergency department
ICU: intensive care unit
PIV: peripheral intravenous
US: ultrasound
